# The impact of selection bias in randomized multi-arm parallel group clinical trials

**DOI:** 10.1371/journal.pone.0192065

**Published:** 2018-01-31

**Authors:** Diane Uschner, Ralf-Dieter Hilgers, Nicole Heussen

**Affiliations:** 1 Department of Medical Statistics, RWTH Aachen University, Aachen, Germany; 2 Center of Biostatistics and Epidemiology, Department of Evidence Based Medicine, Sigmund Freund University, Vienna, Austria; University Hospital Jena, GERMANY

## Abstract

The impact of selection bias on the results of clinical trials has been analyzed extensively for trials of two treatments, yet its impact in multi-arm trials is still unknown. In this paper, we investigate selection bias in multi-arm trials by its impact on the type I error probability. We propose two models for selection bias, so-called *biasing policies*, that both extend the classic guessing strategy by Blackwell and Hodges. We derive the distribution of the *F*-test statistic under the misspecified outcome model and provide a formula for the type I error probability under selection bias. We apply the presented approach to quantify the influence of selection bias in multi-arm trials with increasing number of treatment groups using a permuted block design for different assumptions and different biasing strategies. Our results confirm previous findings that smaller block sizes lead to a higher proportion of sequences with inflated type I error probability. Astonishingly, our results also show that the proportion of sequences with inflated type I error probability remains constant when the number of treatment groups is increased. Realizing that the impact of selection bias cannot be completely eliminated, we propose a bias adjusted statistical model and show that the power of the statistical test is only slightly deflated for larger block sizes.

## Introduction

Multi-arm clinical trials have been gaining more and more importance, particularly due to the recent advances in small population group research [1]. Multi-arm clinical trials often compare multiple experimental treatment arms and a single control arm. They can therefore reduce the sample size in comparison to separate trials with one experimental and one control arm each and increase the willingness of participants to enter the trial [2]. The benefits of multiarm trials are particularly important for very small trials in orphan diseases [3].

Many researchers consider fixed randomization with equal allocation ratio, such as the permuted block design, as the gold standard for allocating patients to multiple treatment groups [4]. However, as blinding in multi-arm randomized controlled clinical trials can be challenging [2], multi-arm randomized trials like the STAMPEDE trial [5] are commonly conducted as open-label studies. Multi-arm trials can therefore be particularly susceptible to *selection bias*, a bias that can be introduced in a clinical trial due to heterogeneity of the patient population resulting from the predictability of the randomization sequence [6]. Even if a randomized trial is conducted double blind, selection bias may be introduced due to unmasking of past treatment assignments, for example due to side-effects. It interferes with the unbiased comparison of treatment effects that is the heart of each randomized controlled clinical trial. Six decades ago, D. Blackwell and J. L. Hodges remarked [7]

It is widely recognized that experiments intended to compare two or more treatments may yield biased results if the experimental subjects are selected with knowledge of the treatments they are to receive.

Since then, the impact of selection bias in randomized clinical trials has been the subject of papers and guidelines [7–17]. Blackwell and Hodges [7] were the first to present a formal approach for quantifying selection bias. Under the assumption that the investigator wishes to make one of the treatments appear better than the other, they presumed that the investigator would try to guess the treatment assignment for the next patient based on the knowledge of the past assignments. For example, he would guess that a treatment is likely to be allocated next when it has so far been allocated less frequently. As a consequence, the investigator would include a patient with better prognosis always when his favoured treatment has currently been allocated less frequently in the trial. A model for the guess of the investigator is called a *guessing strategy*. It has been shown to be an analogue to the degree of the predictability of a randomization sequence based on the allocation probabilities [6]. Strikingly, despite mentioning that selection bias is a problem also in multi-arm clinical trials, all of the mentioned sources focus on two-armed trials. Some researchers may even feel that selection bias disappears when the number of treatment groups increases. In particular, no measure for selection bias in multi-arm randomized controlled clinical trials has been formally introduced. Although Berger et al. [16] conducted a simulation study of the susceptibility of three-armed trials to selection bias, they never formally defined a measure of selection bias for multi-armed trials. Of all the measures that have been proposed for two-arm trials, the impact of selection bias on the type I error probability is most important from a regulatory point of view, as stated for example in the ICH E9 guideline [17].

In the present paper, we propose to measure selection bias in multi-arm trials by its influence on the test decision of the global *F*-test, when selection bias is modeled using a *biasing policy*, a generalization of the guessing strategy for two-arm trials proposed by Blackwell and Hodges [7] that models the heterogeneity in the patient stream due to selection bias. The outline of the paper is as follows. In the section entitled “Model”, we present our assumptions for the outcome model and introduce the permuted block design, the randomization procedure most frequently used for assigning patients to multiple treatment groups. The results are presented in the subsequent section entitled “The Impact of Selection Bias”. There, we generalize the guessing strategy proposed by Blackwell and Hodges [7]. The variability encountered in multi-arm trials admits different extensions. We therefore present two generalized biasing policies that appear plausible in multi-arm trials from a practical point of view. Then we derive the distribution of the *F*-statistic under the misspecified model and present a formula for the exact type I error probability conditional on a randomization sequence, followed by a numerical comparison of the impact of selection bias in multi-arm trials. In the Section entitled “Adjusting for Selection Bias”, we present a selection bias adjusted analysis strategy that can serve as a sensitivity analysis. We conclude with a “Discussion” section. The supporting information contains R code for the computation of the presented formulae.

## Model

Consider a randomized single center clinical trial without interim analyses. Assume patients are allocated using a *K*-arm parallel group design and balanced sample size per group and that the response is a continuous normal outcome. To use formal notation, let the outcome *y*_*i*_ of a patient *i* be the realization of a normally distributed random variable *Y*_*i*_ with mean *μ*_*k*_ if patient *i* is allocated to group *k*, and unknown variance *σ*^2^. Let *N* denote the total sample size and *K* the number of treatment groups.

Usually the situation is embedded in a linear model with one fixed factor

$$\begin{array}{c} y=X\beta +\epsilon , \end{array}$$ (1)

where ***y*** = (*Y*_1_, …, *Y*_*N*_)^*t*^ is the outcome vector, $X\in R^{N\times K}$ the design matrix, $\beta ={\left(\mu_{1},\ldots ,\mu_{K}\right)}^{t}\in R^{K}$ the unknown parameter, and $\epsilon ∼N(0,\sigma^{2}I_{N})$ the normally distributed residual error. The matrix $I_{N}\in R^{N\times N}$ denotes the identity matrix of dimension *N*. In what follows, we consider the null hypothesis that all group means are equal,

$$\begin{array}{c} H_{0}:\mu_{1}=\cdots =\mu_{K}. \end{array}$$ (2)

Under the normal assumption, this hypothesis is usually tested using an *F*-test with test statistic

$$\begin{array}{c} S_{F}=\frac{\frac{1}{K-1}\;y^{t}\left(X{\left(X^{t}X\right)}^{-1}X^{t}-\frac{1}{N}1_{N\times N}\right)\;y}{\frac{1}{N-K}\;y^{t}\left(I-X{\left(X^{t}X\right)}^{-1}X^{t}\right)\;y}, \end{array}$$ (3)

where the matrix $1_{N\times N}\in R^{N\times N}$ has all elements equal to one, and ***X***^*t*^ denotes the transpose of the design matrix ***X***.

The design matrix ***X*** = (*x*_*ik*_) has elements *x*_*ik*_ corresponding to the treatment allocation, namely

$$\begin{array}{c} x_{ik}=\{\begin{array}{cc} 1 & \;\text{if}\;\text{patient}\;i\;\text{is}\;\text{allocated}\;\text{to}\;\text{treatment}\;\text{group}\;k\\ 0 & \;\text{else.} \end{array} \end{array}$$ (4)

As only one treatment is assigned per patient, the sum of each row equals one. The number of patients allocated to each treatment group is given by the sum of the columns ***x***_*k*_ = (*x*_1*k*_, …, *x*_*Nk*_). Obviously, the explicit form of the design matrix is a unique representation of the randomization list resulting from a particular randomization procedure. In the following, we restrict the consideration to fixed sample, non-adaptive, unstratified randomization procedures. We focus our attention on the permuted block design (PBD), the most commonly used randomization procedure for randomized controlled clinical trials with multiple treatment arms. Using the permuted block design, the patient stream is divided into *M* blocks. In each block, the same number of patients *c* is allocated to each of the *K* treatment groups, so that there are *c* ⋅ *K* in each of the *M* blocks. Throughout this article we assume that the last block is complete, so that the total sample size is a multiple of the block length, namely *N* = *c* ⋅ *K* ⋅ *M*. This is a generalization of the blocked design using the notation of Berger et al. [16]. We denote the permuted block design with blocks of length *c* ⋅ *K* by PBD(*cK*). An allocation sequence produced by PBD(*cK*) will necessarily be balanced after each *c* ⋅ *K* allocations. In case of PBD(*K*), the design is balanced after every *K*th patient. As we have *c* = 1, in every block exactly one patient is allocated to each group. In case of PBD(*N*/2), the design is balanced after *N*/2 patients. That means we have two blocks of length *N*/2 and in each block $c=\frac{N}{2K}$ patients are allocated to each treatment group. In case of PBD(*N*), we have one block of length *N* and balance is forced only at the end of the trial. The design PBD(*N*) is also called *random allocation rule* and denoted by RAR.

## Impact of selection bias

The restrictions imposed by the permuted block design introduce a certain predictability of the randomization sequence. This predictability can lead to biased trial results. Already imperfect knowledge of the random assignments, e.g. when some past assignments were unmaksed due to side-effects, is sufficient to make future allocations predictable. Formally, we will characterize predictability by the following two assumptions.

**Assumption 1**. *Past assignments x*_1,*k*_, … *x*_*i*−1, *k*_ *to each treatment group k are unmasked before including patient i*, *so that the number of past assignments to each group*

$$\begin{array}{c} N_{k}\left(i-1\right)=\sum_{j=1}^{i-1}x_{jk}, \end{array}$$

*is known for all treatment groups k* ∈ {1, …, *K*} *and patients i* ∈ {1, …, *N*}. *For i* = 1, *we define N*_*k*_(*i* − 1) = *N*_*k*_(0) = 0 *for all k* = 1, …, *K*.

**Assumption 2**. *In expectation the same number of patients is assigned to all treatment groups*, *namely*

$$\begin{array}{c} E\left(N_{1}\left(N\right)\right)=\cdots =E\left(N_{K}\left(N\right)\right)=N/K. \end{array}$$

Based on these assumptions of predictability, Blackwell and Hodges [7] proposed to model the influence of selection bias on the expected responses in a two-arm trial. They motivate their model by imagining an investigator who wishes to make one of the two treatments appear better than the other, even though the null hypothesis is true. They assume that the investigator, consciously or unconsciously, favours one treatment, say the experimental treatment. If the investigator can guess that the next treatment to be assigned will be the experimental treatment, he might select a patient with higher expected response to be included in the trial. On the other hand, if he guesses the next assignment to be to the other treatment group, he might include a patient with worse expected response. As a particular *guessing strategy*, it is sensible for the investigator to guess the treatment which at that point of the enrollment has been allocated less frequently, knowing that, in the end of the trial, the treatment groups are expected to be balanced. Of course, the situation that an investigator guesses the next treatment assignments constitutes a worst case scenario.

While Blackwell and Hodges [7] where concerned with the impact of selection bias on the mean difference between the treatment groups, we want to measure its impact in hypothesis tests with multi-arm trials. In two-arm trials, Proschan [11] and Kennes et al. [14] showed for the *z*-test and *t*-test respectively that selection bias can seriously inflate the type *I* error rate, when the guessing strategy is incorported in the model of the patients responses. Proschan [11] coined the term *biasing policy* for the model of the biased patients responses.

The generalization of the guessing strategy to multi-arm trials is not straight forward. On the one hand, an investigator might not strictly favour one treatment over all others, but might have a set of favoured treatments F⊂{1,…,K}. On the other hand, ties in the number of patients per treatment group will occur frequently, and there are several options of how to deal with them. In the following, we therefore propose two biasing policies that seem relevant from a practical point of view.

## Biasing policies

A biasing policy is a model for the influence of the guessing strategy on the patients’ responses. Generalizing our model in Eq 1 to include an additional *selection bias effect* η∈R and a *bias vector* ***b*** = (*b*_1_, …, *b*_*N*_)^*t*^, we assume that the patient responses follow the model

$$\begin{array}{c} y=X\beta +\eta b+\epsilon . \end{array}$$ (5)

In what follows, we consider the case where larger values of ***y*** are assumed to be better responses to treatment, and assume *η* > 0 to reflect the physician’s preference for patients with higher expected response. Values of *η* < 0 correspond to a preference for patients with lower expected response. The components of ***b*** are determined by the guessing strategy of the investigator and denote whether the investigator wishes to include a patient with worse (*b*_*i*_ = −1), neutral (*b*_*i*_ = 0), or better (*b*_*i*_ = 1) expected response. Different models for ***b*** arise depending on the guessing strategy of the investigator. The parameter η∈R is the strength of the shift introduced by the investigator. We are interested in the effect of fitting the model described in Eq 1, knowing that due to the misspecification that results from ignoring *η* ***b***, the error term now follows a normal distribution with expectation *η* ***b*** and variance *σ*^2^ ***I***_*N*_.

To determine the components of ***b***, a reasonable generalization of the Blackwell and Hodges model is that the investigator would favour a subset F⊂{1,…,K} of treatment groups, and would assume that any of them will be assigned next, when *all* of the groups in F have fewer patients than the remaining groups. In other words, the investigator will include a patient with better expected response (*b*_*i*_ = 1), if the largest of his favoured groups F has fewer patients than any of the not favoured groups $F^{C}$:

$$\begin{array}{c} \max_{j\in F}N_{j}\left(i-1\right)<\min_{k\in F^{C}}N_{k}\left(i-1\right). \end{array}$$

We say that a group *j* is *larger* than a group *k* at the time of enrollment of patient *i*, if more patients had been enrolled to group *j* than to group *k* prior to the enrollment of patient *i*, so that *N*_*j*_(*i* − 1) > *N*_*k*_(*i* − 1). Conversely, we say that a group *j* is *smaller* than group *k*, if fewer patients have been allocated to group *j*, so that *N*_*j*_(*i* − 1) < *N*_*k*_(*i* − 1)

The investigator will guess that one of the *not* favoured groups will be allocated next, if all of the not favoured groups have fewer patients than the smallest of the favoured groups. In other words, the investigator will include a patient with worse expected response (*b*_*i*_ = −1), if the largest of his not favoured groups is smaller than the smallest of his favoured treatment groups:

$$\begin{array}{c} \min_{j\in F}N_{j}\left(i-1\right)>\max_{k\in F^{C}}N_{k}\left(i-1\right). \end{array}$$

The bias vector in Eq 5 can therefore be modelled with components defined by the following biasing policy.

**Biasing Policy I**: *The components of the bias vector* ***b*** = (*b*_1_, …, *b*_*N*_) *are given by*

bi={-1ifmaxj∈FNj(i-1)<mink∈FCNk(i-1)-1ifminj∈FNj(i-1)>maxk∈FCNk(i-1)-0else. (6)

The following example illustrates that the bias vector depends on the realization of the randomization sequence.

**Example 1**. In a trial with three treatment groups that compares one experimental treatment to two standard of care treatments, the investigator may adopt biasing policy I when he favours the experimental treatment as the favoured treatment, F:={1}. Table 1 shows the computation of the bias vector for the randomization list that is represented by the design matrix ***X*** with the columns ***x***_1_, ***x***_2_, ***x***_3_ shown in the table. We see that the first patient is allocated to group 1, the second to group 2, and so forth. In the beginning (*i* = 1), all groups are balanced, so the investigator includes a patient with neutral response (*b*_1_ = 0). After including the first patient to the experimental group 1, group 1 is larger than any of the standard of care groups 2 and 3. So the investigator will guess that the next patient will be assigned to one of the standard of care groups, and, consequently, include a patient with worse expected response *b*_2_ = −1. After the second patient, the experimental group 1 and the standard of care group 2 have the same number of patients, so the investigator is unsure which treatment will be assigned next, and includes a neutral patient. Continuing this process for the remaining four patients yields the bias vector ***b*** = (0, −1, 0, −1, −1, 0).

**Table 1:** Example for computing the bias vector using biasing policy I in a trial with six patients and three treatment groups (*K* = 3) when the favoured treatment is F={1}.

Patient *i*	*x*_1_	*x*_2_	*x*_3_	*N*_1_(*i* − 1)	*N*_2_(*i* − 1)	*N*_3_(*i* − 1)	*b*_*i*_
1	1	0	0	0	0	0	0
2	0	1	0	1	0	0	−1
3	1	0	0	1	1	0	0
4	0	0	1	2	1	0	−1
5	0	0	1	2	1	1	−1
6	0	1	0	2	1	2	0

An alternate bias model may result in a trial where several doses of an active treatment are compared to a placebo or a control treatment. In this situation the investigator may favour the active treatment, irrespective of the doses. He would try to allocate patients with lower expected response to the control groups, and patient with higher expected response to the experimental groups. Following the same argument as above, the investigator would guess that one of his favoured treatment groups F⊂{1,…,K} will be allocated next, when *any* of the groups in F has fewer patients than any of the treatment groups $F^{C}=\left\{1,\ldots ,K\right\}\setminus F$, and guess the treatment groups $F^{C}$ when any treatment group in F has more patients than the group of F with fewest patients. The patients’s responses can then be modelled according to Eq 5 and the components of the bias vector are defined by the following biasing policy:

**Biasing Policy II**: *The components of the bias vector* ***b*** = (*b*_1_, …, *b*_*N*_) *are given by*

bi={-1ifminj∈FNj(i-1)<mink∈FCNk(i-1)-1ifminj∈FNj(i-1)>mink∈FCNk(i-1)-0else. (7)

As before, the bias vector depends on the randomization sequence, as illustrated in the following example.

**Example 2**. In a trial with three treatment groups, assume that the investigator avoids the placebo treatment ($F^{C}=\left\{1\right\}$) and equally favours the remaining treatment groups (F={2,3}). Table 2 shows the computation of the bias vector for the design matrix *X* given by the columns ***x***_1_, ***x***_2_, ***x***_3_ shown in the table. Note that the design matrix is the same as in Example 1, only the biasing policy changes. The first patient is allocated to the group 1 which is now the not favoured placebo group. After the first allocation, the treatment group 3 is always smaller than the placebo group. Guessing that the next patient will be allocated to group 3, the investigator would include a patient with better expected response. This yields the bias vector ***b*** = (0, 1, 1, 1, 1, 1).

**Table 2:** Example for computing the bias vector using biasing policy II in a trial with six patients and three treatment groups (*K* = 3) when the favoured treatments are F={2,3}.

Patient *i*	*x*_1_	*x*_2_	*x*_3_	*N*_1_(*i* − 1)	*N*_2_(*i* − 1)	*N*_3_(*i* − 1)	*b*_*i*_
1	1	0	0	0	0	0	0
2	0	1	0	1	0	0	1
3	1	0	0	1	1	0	1
4	0	0	1	2	1	0	1
5	0	0	1	2	1	1	1
6	0	1	0	2	1	2	1

Examples 1 and 2 show that biasing policy I may introduce bias for fewer patients than biasing policy II, and can therefore be considered stricter.

### Calculation of type *I* error probability under misspecification

When applying the global *F*-test in the misspecified model given in Eq 1, the type *I* error probability may be biased by the selection bias policy. In order to measure the impact of selection bias on the test decision, we have to derive the distribution of the *F*-statistic *S*_*F*_ in Eq 3 when selection bias is present. When the responses are influenced by selection bias which is defined by the bias vector ***b*** and depends on the randomization sequence, the error term in Eq 1 follows a normal distribution $N(\eta b,\sigma^{2}I)$ that is no longer identically distributed.

We now show that *S*_*F*_, the test statistic of the *F*-test, follows a doubly noncentral *F*-distribution. Using the notation

$$\begin{array}{c} S_{F}=\frac{\frac{1}{K-1}\;y^{t}\left(X{\left(X^{t}X\right)}^{-1}X^{t}-\frac{1}{N}1_{N\times N}\right)\;y}{\frac{1}{N-K}\;y^{t}\left(I-X{\left(X^{t}X\right)}^{-1}X^{t}\right)\;y}\equiv \frac{y^{t}Ay}{y^{t}By} \end{array}$$ (8)

and definition (30.1) of [18], it suffices to show that the quadratic forms ***y***^*t*^ ***Ay*** and ***y***^*t*^ ***By*** are noncentrally *χ*^2^-distributed and stochastically independent. Using Theorem 7.3. of Searle [19], a quadratic form ***y***^*t*^ ***Ay*** with $y∼N(\mu ,\sigma^{2}I)$ is noncentrally *χ*^2^-distributed with *d*_1_ = rank(***A***) degrees of freedom and noncentrality parameters ***μ***^*t*^ ***Aμ*** if the matrix ***A*** is idempotent. In the case of the numerator of Eq 8, the quadratic form is given by ***y***^*t*^ ***Ay*** with

$$\begin{array}{c} A=X{\left(X^{t}X\right)}^{-1}X^{t}-\frac{1}{N}1_{N\times N}. \end{array}$$

Right multiplication of ***X***(***X***^*t*^ ***X***)^−1^ ***X***^*t*^ with the column vector $1_{N}={\left(1,\ldots ,1\right)}^{t}\in R^{N}$ shows that $X{\left(X^{t}X\right)}^{-1}X^{t}\cdot \frac{1}{N}1_{N\times N}=\frac{1}{N}1_{N\times N}$. Hence, *A*^2^ = *A*, so ***A*** is idempotent and ***y***^*t*^ ***Ay*** is noncentrally *χ*^2^-distributed with *K* − 1 degrees of freedom and noncentrality parameter λ_1_ = *η*^2^ ***b***^*t*^ ***Ab***. Similarly, the quadratic form ***y***^*t*^ ***By*** in the denominator of Eq 8 is given by

$$\begin{array}{c} B=I-X{\left(X^{t}X\right)}^{-1}X^{t}. \end{array}$$

Again through multiplication of ***X***(***X***^*t*^ ***X***)^−1^ ***X***^*t*^ with **1**_*N*_ we can show that ***B*** is idempotent and has rank(***B***) = *N* − *K*. Thus, ***y***^*t*^ ***By*** is noncentrally *χ*^2^-distributed with *N* − *K* degree of freedom and noncentrality parameters λ_2_ = *η*^2^ ***b***^*t*^ ***Bb***. Third, using Theorem 7.4 of Searle [19], the quadratic forms are stochastically independent if ***AB*** = 0. This follows directly by multiplication.

In conclusion, *S*_*F*_ follows a doubly noncentral *F*-distribution with *K* − 1 and *N* − *K* degrees of freedom and noncentrality parameters

$$\begin{array}{c} \lambda_{1}=\lambda_{1}\left(b\right)=\eta^{2}b^{t}Ab=\eta^{2}(\frac{1}{n}\sum_{k=1}^{K}{\left(b^{t}x_{k}\right)}^{2}-\frac{1}{N}{\left(b^{t}1\right)}^{2}) \end{array}$$ (9)

and

$$\begin{array}{c} \lambda_{2}=\lambda_{2}\left(b\right)=\eta^{2}b^{t}Bb=\eta^{2}(b^{t}b-\frac{1}{n}\sum_{k=1}^{K}{\left(b^{t}x_{k}\right)}^{2}). \end{array}$$ (10)

Here ***x***_*k*_ denotes the *k*-th column of the design matrix ***X*** formed by the realized randomization list and thus contains all allocations to treatment arm *k* only. From Eqs 9 and 10 it becomes clear that the noncentrality parameters, and therefore the distribution of the test statistic, depends on the particular realization of the randomization sequence. Under the null hypothesis given in Eq 2, the true type I error probability given the design matrix ***X*** corresponding to a particular randomization sequence can be calculated by

$$\begin{array}{c} r\left(X\right)=F_{K-1,N-K}\left(\lambda_{1},\lambda_{2}\right)(|S_{F}|\geq F_{K-1,N-K,1-\alpha }^{-1}), \end{array}$$ (11)

where *F*_*K*−1, *N*−*K*_(λ_1_, λ_2_)(*x*) denotes the distribution function of the doubly-noncentral *F*-distribution with with *K* − 1 and *N* − *K* degrees of freedom and noncentrality parameters λ_1_, λ_2_, and $F_{K-1,N-K,1-\alpha }^{-1}$ denotes the 1 − *α* quantile of the central *F*-distribution. Johnson et al. [18] also give a representation of the conditional cumulative distribution function of *S*_*F*_, see formula (30.51) which can be used for numerical implementation.

We further propose to consider the probability of an inflated type I error probability as evaluation criterion:

$$\begin{array}{c} p_{\text{infl}}\equiv \sum_{X\in \Omega_{PBD}}P\left(X\right)\cdot I\left(r\left(X\right)>\alpha \right), \end{array}$$ (12)

where *P*(***X***) denotes the probability of a randomization sequence represented by ***X***, and *Ω*_*PBD*_ denotes the set of all randomization sequences produced by PBD(*cK*). Further let *I*(*x* > 0.05)≡1 if *x* > 0.05 and *I*(*x* > 0.05)≡0 otherwise. This metric clearly reflects the regulatory viewpoint [17] to maintain the significance level, resulting in a target value of *p*_infl_ = 0.

### Numerical results

This section illustrates the use of the above derivations with numerical examples. We have shown that the rejection probability can be calculated for each individual randomization list generated by the a randomization procedure. However, the number of sequences grows exponentially in *N* and *K*. Therefore, simulations are used for the calculation of the randomization lists, but not for the type I error probability. The derived distribution is represented by box plots and the corresponding summary statistic. In each of the below settings we generate a Monte Carlo sample of *r* = 10,000 randomization sequences for the randomization procedures PBD(*N*), PBD(*K*) and PBD(*N*/2). The number of groups *K* and the number of patients per group *m* = *N*/*K* is varied. The R package randomizeR version 1.3 [20] is used for the generation of the sequences. Then we calculate the distribution of the type I error probabilities as indicated in Eq 11, and the proportion of sequences that lead to an inflated type I error probability as in Eq 12. The selection effect *η* is assumed to be a fraction *η* = *ρ* ⋅ *f*_*m*,*K*_ of Cohen’s effect size *f*_*m*,*K*_ that corresponds to a significance level *α* = 0.05 and a power of 1 − *β* = 0.8. We assume *ρ* ∈ {0, 1/4, 1/2, 1} to investigate the influence of the strength of the bias on the results. In doing so, we adopt a recommendation of Tamm et al. [15] who proposed a similar approach for two-arm trials.

**Fig 1:**
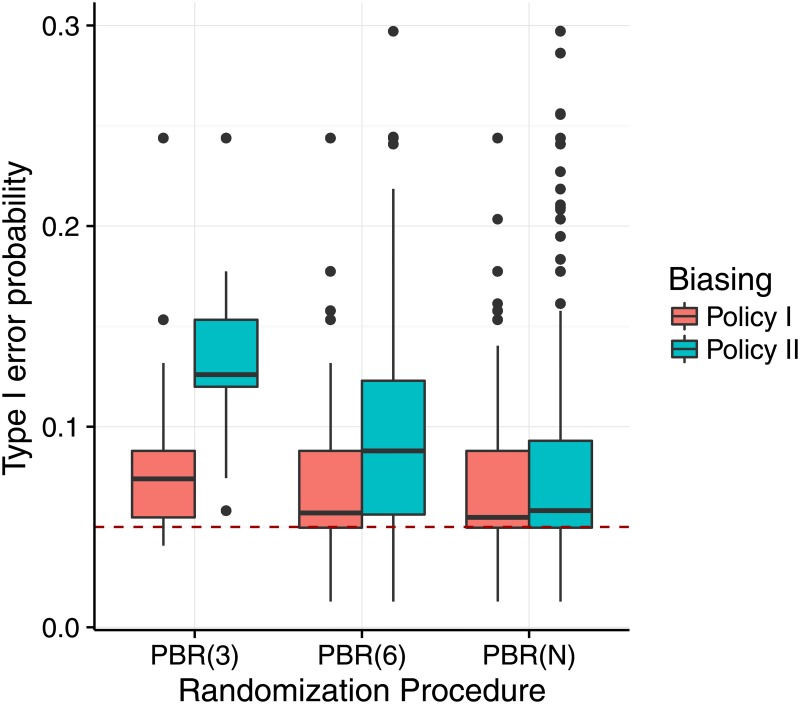
Distribution of the type I error probability under selection bias for different biasing policies. Each scenario is based on a sample of *r* = 10,000 sequences, sample size *N* = 12 and number of treatment groups *K* = 3, assuming the selection effect *η* = *f*_4,3_ = 1.07 for permuted block design (PBD). The red dashed line marks the 5% significance level.

In a first step, the above methodology is applied to investigate the difference between the biasing policies assuming the scenarios of Examples 1 and 2. We set the favoured treatment groups to be $F_{1}=\left\{1\right\}$ for biasing policy I and $F_{2}=\left\{2,3\right\}$ for biasing policy II. We assume an selection effect of *η* = *f*_4,3_ = 1.07. Fig 1 shows the result of the comparison for the sample size *N* = 12 based on the distribution of the type I error probabilities following Eq 11. It can be seen that the distribution of the type I error probabilities is shifted away from the nominal significance level of 5% in all investigated settings. In case of a single block of length *N* (PBD(*N*)), the influence of the biasing policies was comparable. For smaller block sizes, biasing policy II leads to higher type I error probabilities than the biasing policy I.

**Fig 2:**
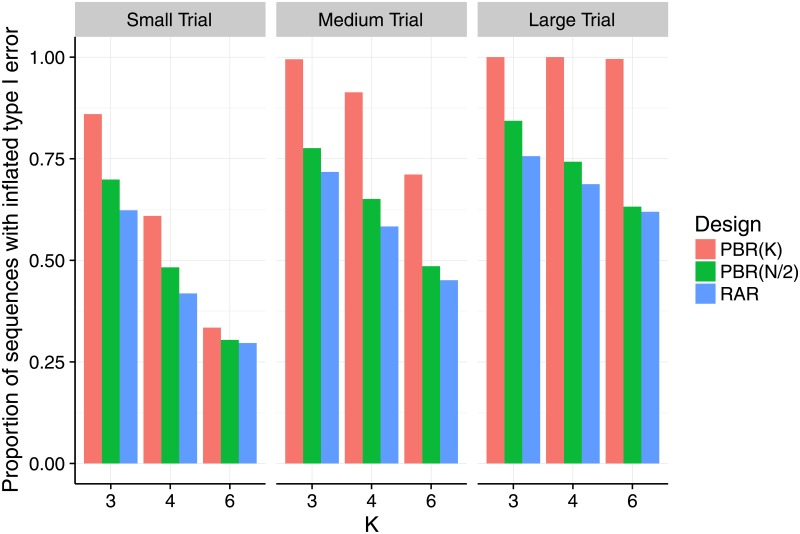
Proportion of sequences that inflate the type I error probability under selection bias for an increasing number of treatment groups, and different block and sample sizes. Each scenario is based on a sample of *r* = 10,000 sequences, assuming the selection effect *η* = *f*_*m*,*K*_ equal Cohen’s size *f*_*m*,*K*_, which depends the group size *m* = *N*/*K* (small: *m* = 4, medium: *m* = 8, large: *m* = 32), and on the number of treatment groups *K* ∈ {3, 4, 6}.

**Fig 3:**
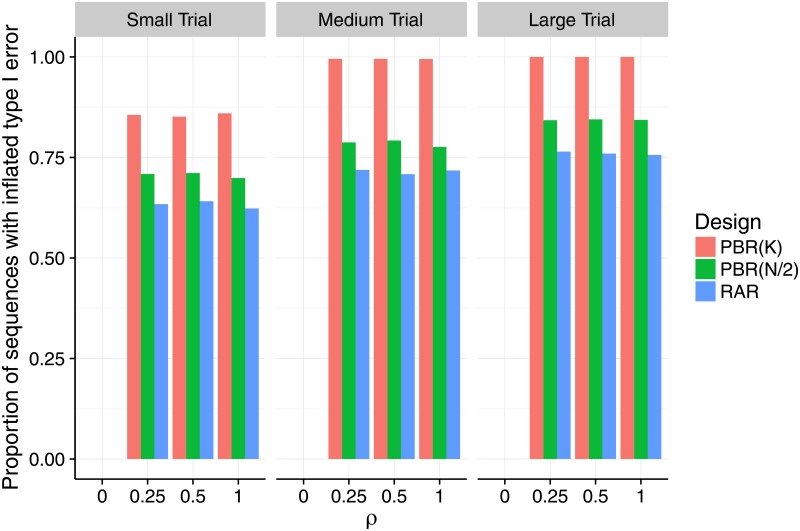
Proportion of sequences that inflate the type I error probability under selection bias for increasing selection effect, and different block and sample sizes. Each scenario is based on a sample of *r* = 10,000 sequences, assuming the selection effect *η* = *ρ* ⋅ *f*_*m*,*K*_ to be a proportion *ρ* of the Cohen’s size *f*_*m*,*K*_, which depends on the group size *m* = *N*/*K* (small: *m* = 4, medium: *m* = 8, large: *m* = 32), and the number of treatment groups which are fixed at *K* = 3. The selection effect *η* increases as *ρ* ∈ {0, 1/4, 1/2, 1}.

In the second step, we restricted our attention to the strict biasing policy with F={1} to investigate the impact of selection bias under variation of the number of groups, the sample size and the selection effect. To that aim, we varied the number of treatment groups *K* ∈ {3, 4, 6} and the number of patients per group *m* = *N*/*K* ∈ {4, 8, 32}, speaking of a small trial if *m* = 4, a medium trial if *m* = 8, and a large trial if *m* = 32. Figs 2 and 3 show the proportion of sequences that lead to an inflation of the type I error probability as proposed in Eq 12. In Fig 2 we fixed the selection effect *η* = *f*_*m*,*K*_, but varied *K* and *m*. In Fig 3 we fixed the number of groups at *K* = 3, but varied *η* = *ρ* ⋅ *f*_*m*,*K*_ and *m*. In all scenarios we investigated, at least thirty percent of the sequences in the sample lead to an inflation of the type I error-probability. However, the maximum proportion of inflated sequences varied according to the randomization procedure. The permuted block design with block size *K* had up to 100% of inflated sequences in medium and large trials (middle and right hand panels of Figs 2 and 3). For permuted block randomization with block length *N*/2 or *N*, the proportion of inflated sequences ranged up to 84% right hand panel of Fig 3 and 76% middle panel of Fig 3 and generally attained its maximum in large trials with *K* = 3 treatment groups. For all the randomization procedures we investigated, the proportion of inflated sequences grew when the number of treatment groups remained the same but the number of patients per group was increased. Consider for example the situation of *K* = 6 treatment groups and permuted block design with block length *K* shown in red in Fig 2. In a small trial, one third of the sequences had inflated type I error probability. This proportion was more than doubled in a medium trial (71%), and reached 100% in a large trial. Interestingly, Fig 3 shows that the proportion of sequences with inflated type I error probability remained constant when the selection effect *η* = *ρ* ⋅ *f*_*m*,*K*_ was varied with *ρ* ∈ {0, 1/4, 1/2, 1} and the number of groups was fixed to *K* = 3. This means that already a relatively small bias can lead to the same proportion of sequences with inflated type I error probability as a large bias. Table 3 shows that this is also true for *K* = 4 and *K* = 6. For *η* = *ρ* = 0, all sequences maintain the type I error in all investigated scenarios, as expected.

**Table 3:** Proportion of sequences with inflated type I error probability. Calculations are based on Eq 12. We set the significance level *α* = 0.05 and the selection effect *η* = *ρ* ⋅ *f*_*m*,*K*_, where *f*_*m*,*K*_ denotes Cohen’s effect size, *K* the number of treatment groups and the number of subjects per group *m* = *N*/*K*.

		*m* = 4			*m* = 8			*m* = 32		
		PBR(K)	PBR(N/2)	PBR(N)	PBR(K)	PBR(N/2)	PBR(N)	PBR(K)	PBR(N/2)	PBR(N)
*K* = 3	*ρ* = 0	0.000	0.000	0.000	0.000	0.000	0.000	0.000	0.000	0.000
	*ρ* = 0.25	0.856	0.709	0.634	0.995	0.787	0.719	1.000	0.843	0.764
	*ρ* = 0.5	0.851	0.711	0.641	0.995	0.792	0.708	1.000	0.845	0.760
	*ρ* = 1	0.860	0.699	0.623	0.995	0.776	0.718	1.000	0.843	0.756
*K* = 4	*ρ* = 0	0.000	0.000	0.000	0.000	0.000	0.000	0.000	0.000	0.000
	*ρ* = 0.25	0.612	0.498	0.422	0.919	0.660	0.591	1.000	0.752	0.695
	*ρ* = 0.5	0.621	0.494	0.422	0.917	0.656	0.601	1.000	0.753	0.689
	*ρ* = 1	0.609	0.483	0.418	0.913	0.651	0.583	1.000	0.743	0.687
*K* = 6	*ρ* = 0	0.000	0.000	0.000	0.000	0.000	0.000	0.000	0.000	0.000
	*ρ* = 0.25	0.345	0.302	0.326	0.711	0.498	0.440	0.996	0.628	0.615
	*ρ* = 0.5	0.344	0.307	0.314	0.702	0.482	0.458	0.998	0.637	0.603
	*ρ* = 1	0.334	0.304	0.296	0.711	0.485	0.451	0.996	0.632	0.619

**Fig 4:**
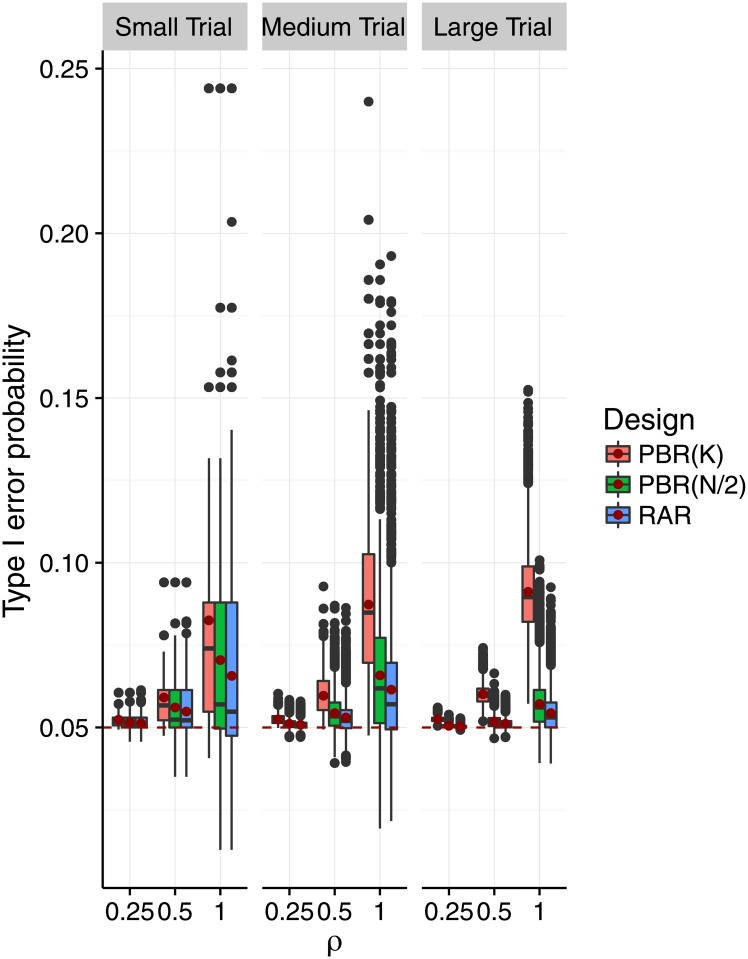
Distribution of the type I error probability under selection bias for increasing selection effect, and different block and sample sizes. Each scenario is based on a sample of *r* = 10,000 sequences, assuming the selection effect *η* = *ρ* ⋅ *f*_*m*,*K*_ to be a proportion *ρ* of the Cohen’s size *f*_*m*,*K*_, which depends on the group size *m* = *N*/*K* (small: *m* = 4, medium: *m* = 8, large: *m* = 32), and the number of treatment groups which are fixed at *K* = 3. The selection effect *η* increases as *ρ* ∈ {0, 1/4, 1/2, 1}. A red dot marks the mean type I error probability in each scenario. The red dashed line marks the 5% significance level. The axis range is (0, 0.25).

**Fig 5:**
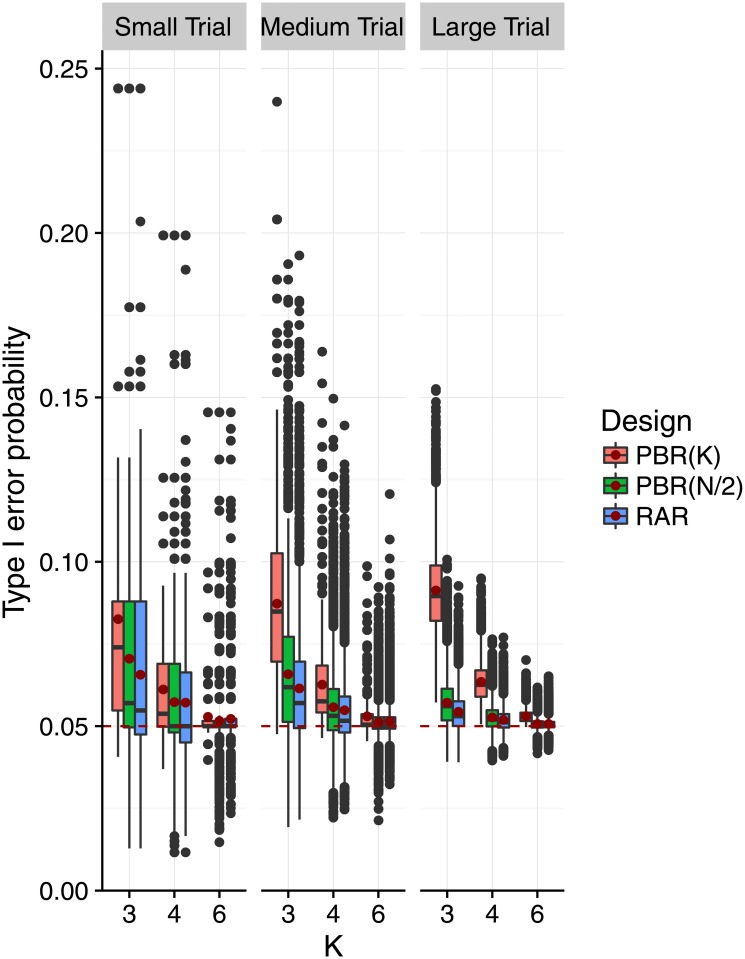
Distribution of the type I error probability under selection bias for an increasing number of treatment groups, block and sample sizes. Each scenario is based on a sample of *r* = 10,000 sequences, assuming the selection effect *η* = *f*_*m*,*K*_ equal Cohen’s size *f*_*m*,*K*_, which depends the group size *m* = *N*/*K* (small: *m* = 4, medium: *m* = 8, large: *m* = 32), and on the number of treatment groups *K* ∈ {3, 4, 6}. A red dot marks the mean type I error probability in each scenario. The red dashed line marks the 5% significance level. The axis range is (0, 0.25).

Figs 4 and 5 show the impact of selection bias on the distribution of the type I error probabilities as proposed in Eq 11. In Fig 4, we varied the selection effect *η* = *ρ* ⋅ *f*_*m*,*K*_ for fixed *K* = 3, and in Fig 5 we varied *K* while fixing *η* = *f*_*m*,*K*_. We can see in Fig 4 that both the variability and mean of the type I error probability increased with increasing selection effect. This effect is less pronounced in medium and large trials than in small trials. The shift of mean and median was most pronounced for block size *K*. As pictured in Fig 5, the variability of the type I error probabilities decreased with the number of treatment groups when the selection effect is *η* = *f*_*m*,*K*_. Also, the mean of the type I error probabilities approaches the 5% significance level. Given a number of treatment groups *K*, the variability decreased with the size of the trial, while the mean type I error probability remained the same.

## Adjusting for selection bias

In this section, we present a possible unbiased analysis strategy that can serve as a sensitivity analysis. When the response is affected by selection bias as modeled in Eqs 6 or 7, the responses follow the linear model described in Eq 1. In contrast to the previous sections where we investigated the influence of model misspecification on the type *I* error probability, we now want to investigate the influence of fitting the correct model, namely,

$$\begin{array}{c} y=\tilde{X}\tilde{\beta }+\epsilon , \end{array}$$

on the power, where the design matrix contains an additional column that accounts for the bias $\tilde{X}=\left[x_{1},\ldots ,x_{K},b\right]$ and the unknown parameter contains the selection effect as an additional unknown parameter $\tilde{\beta }=\left(\mu_{1},\ldots ,\mu_{K},\eta \right)$. Because we included the selection bias effect *η* in the model, the random error is independently and identically distributed $\epsilon ∼N(0,\sigma^{2}I)$. As before, a global *F*-test can be used to test the null hypothesis of equal expectation in the groups as given in Eq 2. We conducted a simulation study to investigate the performance of this bias adjusted test in a practical scenario.

**Fig 6:**
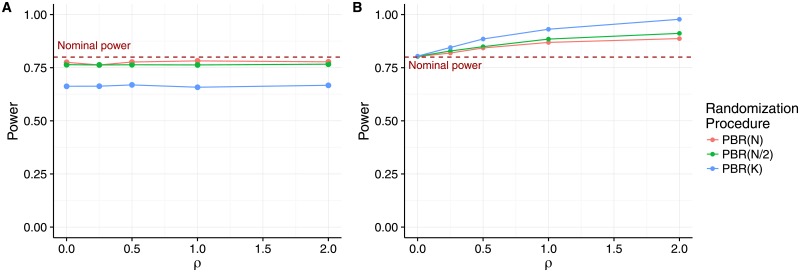
Power of the adjusted test compared to the unadjusted test. A) Power of the *F*-test adjusted for selection bias. B) Power of the *F*-test not adjusted for selection bias. Both panels assume total sample size *N* = 48, *K* = 3 treatment groups and selection effect *η* = *ρ* ⋅ *f*_16,3_ with *ρ* ∈ {0, 0.5, 1, 2}.

Fig 6 shows the power of the bias adjusted *F*-test and, as a reference, the power for the unadjusted *F*-test for the permuted block design with block lengths *N*, *N*/2 and *K*. We assume a sample size of *N* = 48 and *K* = 3 treatment groups, and get an effect size of *f*_*m*,*K*_ = *f*_16,3_ = 0.9829 corresponding to Cohen’s effect size for a power of 80% at significance level *α* = 0.05. We assumed an increasing selection effect *η* = *ρ* ⋅ *f*_16,3_ with *ρ* ∈ {0, 0.5, 1, 2}. We used the R package car [21] to account for the type III sum of squares required due to the unbalanced design induced by the biasing policy.

We can see that the unadjusted *F*-test in panel B keeps the planned power of 80% only if *η* = *ρ* = 0. In all other cases, the presence of selection bias leads to an over-estimation of the treatment difference, resulting in an inflated power increasing with *ρ*. The degree of the inflation depends on the block length, reflecting the predicability of the permuted block design. For all of the block lengths we investgated, the power of the selection bias adjusted test in panel A is constant when *η* = *ρ* ⋅ *f*_*N*, *K*_ increases. The power suffers only slightly from fitting the additional factor in the model when we use PBD(*N*/2) or PBD(*N*). When using PBD(*K*) the power is drastically reduced to about 66%.

Note that this approach also provides a maximum likelihood estimator for the selection effect *η*, and a test for the presence of selection bias, deriving the distribution of the *F*-test statistic under the null hypothesis *H*_0_: *η* = 0. The steps are similar to those of [22] who derived a likelihood ratio test for the presence of selection bias in two-arm trials. We recommend conducting the selection bias adjusted test as a sensitivity analysis for the presence of selection bias.

## Discussion

We have shown that more than two treatment arms do not protect the test decision in a clinical trial from the influence of selection bias. While the extent of the distortion of the test decision may depend on a variety of possible settings, the fact that selection bias can impact the test decision has to be acknowledged also under very conservative assumptions. Contrary to common misconceptions (cf. [16], Sec. 5), we showed that selection bias poses a serious risk even when the number of treatment groups or the sample size is large.

We proposed two biasing policies for selection bias that generalize the guessing strategy that has been proposed for two-arm trials by Blackwell and Hodges [7]. Using these models, we derived a formula for calculation of the impact of selection bias on the overall *F*—test, which can be applied to all non-adaptive, unstratified randomization procedures. We derived the exact conditional distribution of the test statistic given a particular randomization sequence, and proposed a formula for the exact rejection probability given a randomization sequence under the selection bias model. This makes it possible to evaluate the influence of selection bias on the type I error probability, as required by the ICH E9 guideline [17]. In contrast to previous approaches, e.g. [11], the approach we presented not only provides the mean distortion of the type I error rate, but also covers its variability accross randomization sequences. We applied the derivation to quantify the impact of selection bias on the test decision in multi-arm clinical trials with permuted block design. Our results show that previous findings [14, 15, 23] extend to multi-arm clinical trials; namely the influence of selection bias on the mean type I error probability is most pronounced for small block sizes. While the extent of the inflation of the type I error was shown to be sensitive to the biasing policy, small block sizes have been shown to be problematic irrespective of the biasing policy employed. In the investigated scenarios, selection bias lead to an inflation of the power when it was not accounted for in the analysis. Preliminary research shows that this unadjusted test can also lead to a deflation of the power in some scenarios when the variability of the responses outweighs the effect on the estimated treatment effect. We further showed that the adjustment for selection bias in the analysis leads to a substantial loss in power when small block sizes are used. To protect multi-arm trials against selection bias, we recommend that a randomization procedure with very few restrictions should be used. In particular, the permuted block design should only be used with large block sizes. Then a selection bias adjusted test can serve as a sensitivity analysis for the susceptibility of the results to selection bias. Note that, under the Blackwell and Hodges model, random block sizes do not provide any benefit for the reduction of selection bias [6].

We strongly encourage researchers and clinical trialists to assess the extent of selection bias for a variety of block lengths and, if available, randomization procedures at the planning stage of their particular trial. We recommend to follow a procedure similar to the template proposed by Hilgers et al. [24]. In any case, investigators should always report the randomization procedure and the parameters they used according to the CONSORT 2010 statement [25], along with their reasons for choosing the randomization procedure.

The considerations presented in this article are subject to various limitations. To begin with, we restricted the consideration to an equal allocation, non-adaptive, unstratified permuted block design. However, the derivation can directly be applied to unequal allocation ratios and other restricted randomization procedures. As stratification induces balance across strata, we expect that the results will be comparable to those observed in this investigation when stratified randomization is used. The effects of selection bias in covariate- or response-adaptive randomization have not yet been studied in the literature. As their implementation comes with additional complexities, we did not include these randomization procedures here, but concentrated on one of the simplest, most frequently used randomization procedure. Clearly, the settings we chose for the comparative study are quite limited. In particular, we considered only two possible biasing policies. Other biasing policies might lead to other conclusions. The extent of the impact on the type I error probability depends on the number of groups and the sample size. We particularly focused on small sample sizes, motivated by the IDeAl FP7 project that investigated new statistical design and analysis methodologies in small population clinical trials. Even so, the examples we presented offer a general impression, and serve as a motivation for the scientist to conduct his own evaluation using the R package randomizeR [20] and the tools provided in the supplementary material. Lastly, we acknowledge that the assumption of normally distributed outcomes is very restrictive in practice. Other, for example binary, outcomes could be incorporated through the use of generalized linear models that would also admit the adjustment for covariates. However, to our knowledge, this is the first investigation of multi-arm clinical trials with respect to selection bias. Subject to future research should also be the relation of the type I error inflation to other measures for selection bias, such as the predictability of the randomization sequence [6]. Furthermore, the effect of other biases, such as chronological bias caused by time-trends (cf. [26]), should not be neglected in the design and analysis stage of clinical trials.

## Supporting information

S1 File R-Code for the calculation of type *I* error probability under misspecification. The functions contained in this file implement the biasing policies, the non-centrality parameters of the doubly noncentral *F*-distribution, and the rejection probability.
(R) Click here for additional data file.

S2 File R-Code for conducting the simulation study. This code conducts the simulation study that is the basis for Figs 1–5 and Table 3.
(R) Click here for additional data file.

S3 File Simulation settings. This comma seperated values file includes the simulation settings that were the basis for Figs 1–5 and Table 3.
(CSV)

S4 File R-Code for generation of the figures. This file includes the code for generating Figs 1–5 from the results of the simulation study.
(R) Click here for additional data file.

S5 File R-Code for conducting the selection bias adjusted test. This code conducts the simulation study and executes the selection bias adjusted test that is the basis for Fig 6.
(R) Click here for additional data file.
